# Giant synovial cell sarcoma of the thorax in a 46-year-old man: a case report

**DOI:** 10.1186/1757-1626-2-9324

**Published:** 2009-12-15

**Authors:** Saulat Hasnain Fatimi, Taimur Saleem

**Affiliations:** 1 Department of Surgery, Division of Cardiothoracic Surgery, Aga Khan University, (Stadium Road), Karachi, (74800), Pakistan; 2 Medical College, Aga Khan University, (Stadium Road), Karachi, (74800), Pakistan

## Abstract

**Background:**

Although synovial cell sarcoma is a common tumor of the extremities, its occurrence in the thorax has been less frequently documented.

**Case presentation:**

A 46-year-old Pakistani man presented with a 2 month history of progressively increasing cough and left lower chest pain. Initial evaluation was done using a chest x-ray; the patient was found to have a large mass involving the lower portion of the left chest. A computed tomography scan was performed next which showed a large mass involving the left chest wall with invasion into the pericardium and left hemidiaphragm. En bloc surgical resection of the tumor was undertaken. Final pathology showed synovial cell sarcoma of the thorax. At one-year follow-up, the patient has shown no recurrence of the disease.

**Conclusion:**

We have described a rare case of a large synovial cell sarcoma of the thorax. Surgical resection appears an appropriate modus operandi for managing giant synovial cell sarcomas of the thorax. However, there is a need to clearly define post-operative strategies for cases with extensive involvement of surrounding structures.

## Background

Synovial cell sarcoma (SCS) is a common tumor of the extremities. However, SCS of the thorax is an overall rare clinical entity. We report here a case of SCS in a 46-year-old man with extensive involvement of the chest wall, lung and diaphragm.

## Case presentation

**Figure 1:**
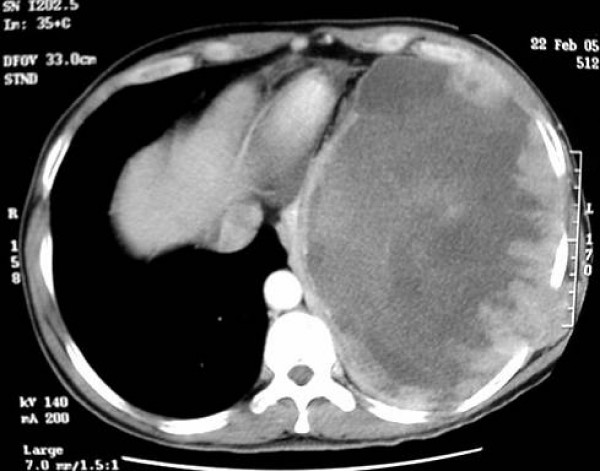
**Computed tomography scan of the chest showing a large synovial cell sarcoma with extensive involvement of surrounding structures**.

A 46-year-old Pakistani man presented to our hospital with a 2 months history of progressively increasing cough and left lower chest pain. Initial evaluation was done using a chest x-ray and he was found to have a large mass involving the lower portion of the left chest. Computed tomography (CT) scan was obtained next to better delineate the disease process (Figure 1). It showed a 16 × 15 cm mass involving the left chest wall and left lower lobe of the lung with invasion into the pericardium and left hemidiaphragm. There was no pericardial or pleural effusion. CT scan of the abdomen-pelvis was normal.

**Figure 2:**
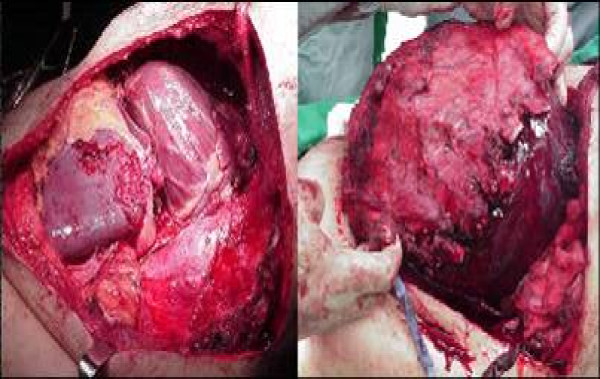
**Large synovial cell sarcoma with extensive involvement of the chest wall, pericardium, left hemidiaphragm and left lower lung lobe**.

Trucut biopsy of this mass showed features of SCS. In the absence of effective chemotherapy and obvious metastasis, the patient was planned for a complete resection of this tumor. The tumor was exposed via a left posterolateral thoracotomy approach. Intra-operatively, a large tumor with extensive involvement of the left lower chest wall from the 4th to 10^th ^ribs was seen. It was also involving the left lower lung lobe, left hemidiaphragm and pericardium (Figure 2). The mass was removed en bloc with all involved ribs, left lower lung lobe, left hemidiaphragm and pericardium. Final pathology confirmed it to be SCS sarcoma with negative histological margins. The post operative course of the patient was unremarkable. He was discharged on the 9th postoperative day. At follow-up after one year, the patient has shown no recurrence of the disease.

## Discussion

SCS is a malignant tumor that has been termed such because of histological resemblance to developing synovium [1]. It is thought to arise from primitive pluripotent mesenchymal cells rather than the synovium [2]. SCS is the third most common sarcoma of the extremities after liposarcoma and malignant fibrous histiocytoma [3] and has been found to account for 5 - 14% of soft tissue sarcomas in different studies [1,4,5]. Apart from the extremities, SCS may also occur in the pericardium, pleura, lung, sternum, esophagus, retroperitoneum, mediastinum, mesentery, head and neck, abdominal and thoracic wall, prostate and kidney; albeit with lesser frequency.

SCS of the thorax represents a diagnostic challenge because of the diverse array of competing diagnosis and rarity of incidence [6]. The symptoms of a patient with SCS of the thorax depend on the structures undergoing compression or invasion from the tumor. Patients may present with chest pain, coughing, hemoptysis, dyspnea, reduced breath sounds and weight loss. Primary thoracic SCS sarcomas typically occur as chest wall masses that exhibit a propensity for invasion into surrounding structures. Immunohistochemically, synovial sarcomas show immunoreactivity for cytokeratins, EMA, S-100 protein, are positive for Bcl-2, O-13, Actin and negative for CD34 and Desmin [7]. Young age, Her-2 expression, complete resection with clear surgical margins and response to first line chemotherapy were found as good prognostic indicators in advanced disease in different studies [1,8,9]. Adverse prognostic factors for SCS include lesions larger than 5 cm, male gender, extensive tumor necrosis, neurovascular invasion, aneuploidy and poor histological differentiation [4,10,11]. Definitive diagnosis of SCS can be made with biopsy and use of immunohistochemistry, electron microscopy and cytogenetic analysis [6,12]. CT scan and magnetic resonance imaging (MRI) are imaging modalities employed for the evaluation of respectability and staging of the neoplasm.

Treatment of choice for SCS sarcomas of the thorax is excision, radiation therapy and adjuvant chemotherapy following resection. In our case, successful surgical resection was performed for a large synovial sarcoma of the left chest wall with involvement of left lung, pericardium and left hemidiaphragm. The diaphragm and left chest wall had to be reconstructed with prolene mesh and muscle flap after complete resection of tumor. Our patient is not receiving any radiation therapy because of proximity of the bare heart to the chest wall. Also, the need for adjuvant therapy was obviated because of achievement of histologically negative margins. As SCS is known to recur, the patient needs to be followed up carefully.

## Conclusion

We have documented a rare case of a giant SCS of the thorax with extensive involvement of surrounding structures. This is a rare clinical entity and there is a need to clearly define post-operative strategies for a case with extensive involvement like ours.

## Abbreviations

CT: computed tomography; MRI: magnetic resonance imaging; SCS: synovial cell sarcoma.

## Consent

Written, informed consent was obtained from the patient for the publication of this case report and accompanying images. A copy of the consent form is available for review by the Editor-in-Chief of the journal.

## Competing interests

The authors declare that they have no competing interests.

## Authors' contributions

TS was involved in data collection, interpretation and drafting the manuscript. SHF was involved in study design and conception, data interpretation, drafting the manuscript and providing overall supervision in the project. All authors read and approved the final manuscript.
